# Activated hepatic stellate cells impair NK cell anti-fibrosis capacity through a TGF-β-dependent emperipolesis in HBV cirrhotic patients

**DOI:** 10.1038/srep44544

**Published:** 2017-03-14

**Authors:** Jijing Shi, Juanjuan Zhao, Xin Zhang, Yongqian Cheng, Jinhua Hu, Yuanyuan Li, Xin Zhao, Qinghua Shang, Yanling Sun, Bo Tu, Lei Shi, Bin Gao, Fu-Sheng Wang, Zheng Zhang

**Affiliations:** 1 Department of Infectious Diseases, 302 Military Hospital of China-Peking University Teaching Hospital, Beijing, 100039, China; 2 Research Center for Clinical & Translational Medicine, Beijing 302 Hospital, Beijing, 100039, China; 3 Department of Infectious Diseases, Beijing 302 Hospital, Beijing, 100039, China; 4 Research Center for International Liver Disease, Beijing 302 Hospital, Beijing 100039, China; 5 Research Center for Liver Failure, Beijing 302 Hospital, Beijing, 100039, China; 6 Forensic Sursery Center, Beijing 302 Hospital, Beijing, 100039, China; 7 Department of Infectious Diseases, 88th Hospital of PLA, Taian, 271000, China; 8 Laboratory of Liver Diseases, National Institute on Alcohol Abuse and Alcoholism, National Institutes of Health, Bethesda, MD 20892, USA

## Abstract

Natural killer (NK) cells can induce liver fibrosis remission by killing hepatic stellate cells (HSCs) and producing interferon (IFN)-γ in a mouse model; however, their anti-fibrotic immune-characteristics and regulatory mechanisms by HSCs remain to be determined, especially in livers from HBV-infected liver cirrhosis (LC) patients. We analyzed frequency, phenotype and anti-fibrotic function of hepatic and peripheral NK subsets in 43 HBV-LC patients. We found that hepatic NK subsets from LC patients displayed a decreased frequency, activation status and anti-fibrotic activity compared with those from chronic hepatitis B patients, which were mainly mediated by increased intrahepatic tumour-growth factor (TGF)-β because blockade of TGF-β significantly reversed NK anti-fibrotic function *in vitro. In vivo*, hepatic NK cells were enriched in proximity to the α-smooth muscle actin (α-SMA+) area within mild fibrosis regions; while in severe fibrotic areas, they were either directly attached to or separated from the α-SMA+ region. NK cells from LC patients could enter HSCs to form emperipolesis (a cell-in-cell structure) and become apoptotic; anti-TGF-β treatment ameliorated this emperipolesis. This finding suggested a novel mechanism by which activated HSCs impair NK cells’ anti-fibrosis capacity through a TGF-β-dependent emperipolesis in LC patients, providing an anti-fibrotic rational by enhancing NK cell activity.

Hepatic fibrosis is a prominent feature of persistent infection by hepatitis B virus (HBV) and hepatitis C virus (HCV), as well as any conditions that repeatedly kill hepatocytes, such as alcohol abuse, repeated administration of hepatotoxic drugs, cholestatic disorders or inherited disorders. Hepatic fibrosis is generally associated with chronic hepatic inflammation and injury. In this process, hepatic stellate cells (HSCs) are activated by inflammatory cytokines and mediators, which is followed by matrix deposition, fibrosis and eventually liver cirrhosis (LC), which has been observed extensively in clinical settings^1^,^2^. Thus, HSCs have assumed a central role in liver fibrogenesis^3^,^4^; however, the relevant mechanisms remain obscure.

The interactions between HSCs and immune cell subsets have emerged as important determinants of liver fibrosis progression and regression^1^,^3^,^4^. It is accepted commonly that Th1 cells suppress fibrosis, whereas Th2 cells promote fibrosis^5^. B cells, previously reported to be involved liver fibrosis progression, generated contradictory results^6^,^7^. Natural killer T- (NKT) cells also play dual roles in controlling HSC activation and liver fibrosis by producing both anti-fibrotic and pro-fibrotic cytokines^8^,^9^. Particularly, natural killer (NK) cells, being abundant in the liver and serving as the major innate immune component against infection and tumours^10^, also play an important role in inhibiting liver fibrosis^11^,^12^. These results were later confirmed in other mouse models of liver fibrosis^13^,^14^,^15^ and in HCV patients by several clinical studies^14^,^16^,^17^. Taken together, these studies suggested that the host could orchestrate highly complex immune responses to regulate liver fibrogenesis during chronic virus infection.

The underlying mechanisms of the interactions between NK cells and HSCs have been investigated widely in recent years, but remain to be illusive. Early-activated HSCs might produce increased amounts of retinoic acid, which elevates RAE-1/NKG2D signals^11^,^18^ or produce MICa^19^, to trigger the killing of HSCs by NK cells. HSCs can also express the NKp46 ligand^14^,^16^,^20^ or the NKp30 ligand^21^, which also causes their killing by NK cells. Upon activation of HSCs, MHC class I production is also downregulated, resulting in the reduced engagement of inhibitory NK cell receptors and enhanced killing^12^,^15^. Inflammatory cytokines can further influence this process. NK cell-derived IFN-γ exerts its anti-fibrotic effects by inducing HSC apoptosis and cell cycle arrest^22^,^23^ or by killing activated HSCs via tumor necrosis factor-related apoptosis-inducing ligand (TRAIL) and fas ligand (FasL)^17^. Collectively, these findings suggested that NK cells are likely to play an important role in alleviating liver fibrogenesis by interacting HSCs. The mechanisms mentioned above provide some insight into the molecular mechanisms of how NK cells kill HSCs and antagonise fibrosis. However, to date, little attention has been paid to the immune characterisation of hepatic NK cells in patients and the impact of HSCs on NK cells during liver fibrogenesis^24^, especially in LC patients with chronic HBV infection. Indeed, the anti-fibrotic function of NK cells can also be suppressed by elevated tumor-growth factor (TGF)-β via downregulation of NKG2D and 2B4 expression during chronic liver injury, thus contributing to the progression of liver fibrogenesis^23^,^25^.

In this study, we characterized the impact of liver cirrhosis on hepatic and peripheral NK cells in HBV-infected patients comprehensively, and investigated the morphological and functional interactions between HSCs and hepatic NK cells in HBV patients. Our results indicated that HSCs not only interact physically with NK cells, but also might contribute to NK cell depletion by cellular emperipolesis mediated by HSCs-derived TGF-β. Our findings will promote the rational development of immunotherapeutic strategies to enhance NK activity while limiting or abolishing liver fibrosis in chronic HBV infection.

## Results

### Hepatic NK cells are reduced and deactivated in HBV-infected LC patients

**Figure 1:**
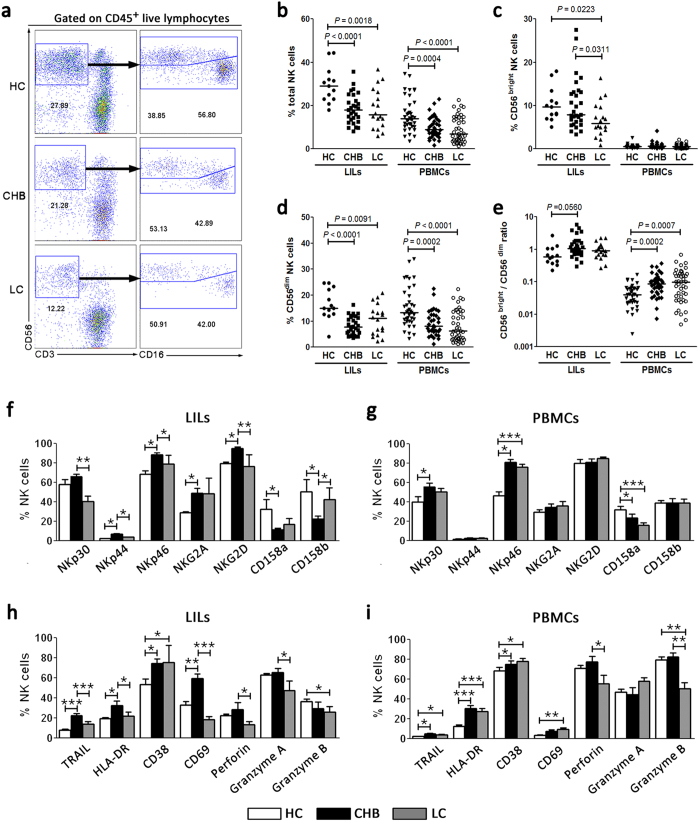
Hepatic NK cells are deactivated in HBV-infected patients with liver cirrhosis. (**a**) Representative dot plots depicting the proportion of total CD3^−^CD56^+^ NK cells and CD56^bright^ and CD56^dim^ NK subsets in liver-infiltrating lymphocytes (LILs) among CHB and LC patients and HC subjects. The numbers indicate the percentages of NK cells within CD45^+^ live lymphocytes (left line) or within CD3^−^CD56^+^ total NK cells (right line). (**b–e**) Pooled data showing the percentages of hepatic and peripheral total NK cells (**b**), CD56^bright^ NK subsets (**c**), CD56^dim^ NK subsets (**d**) and CD56^bright^ and CD56^dim^ ratio (**e**) in the three indicated groups (for liver, n = 13 for HC, n = 29 for CHB, n = 18 for LC; for peripheral blood, n = 38 for HC, n = 36 for CHB, n = 43 for LC). Each dot represents one individual. The horizontal lines indicate the mean values. *P*-values shown in the figures are based on two-tailed, unpaired Student’s t-test. (**f–i**) Summary data show the percentages of hepatic (**f** and **h**) and peripheral (**g** and **i**) CD3^−^CD56^+^ NK cells that express NK receptors, such as NKp30, NKp44, NKp46, NKG2A, NKG2D, CD158a and CD158b (**f** and **g**), and activation and function markers, such as TRAIL, HLA-DR, CD38, CD69, perforin, and Granzyme A and B (**h** and **i**) among the three groups of subjects (for liver, n = 6 for HC, n = 22 for CHB, n = 7 for LC; for peripheral blood, n = 18 for HC, n = 32 for CHB, n = 35 for LC). The data are shown as the means, and the error bars represent the SEM. *P < 0.05, **P < 0.01 and ***P < 0.001, two-tailed, unpaired Student’s t-test.

We first investigated hepatic and peripheral CD3^−^CD56^+^ total NK cells, CD3^−^CD56^bright^ NK and CD3^−^CD56^dim^ NK subset frequency in healthy control (HC), chronic hepatitis B (CHB) and LC subjects using flow cytometry (Fig. 1a). The percentages of hepatic and peripheral total NK cells were reduced significantly in CHB and LC patients compared with the HC subjects (Fig. 1b). Further analysis indicated that the hepatic CD56^bright^ NK subset was reduced preferentially in LC patients compared with CHB and HC subjects. No significant difference in hepatic CD56^bright^ NK subset percentages was observed between CHB and LC patients. The peripheral CD56^bright^ NK subset frequencies were similar in the three groups of subjects (Fig. 1c). By contrast, CD56^dim^ NK subsets from both liver-infiltrating lymphocytes (LILs) and peripheral blood mononuclear cells (PBMCs) displayed a significant decrease in CHB and LC patients compared with those from HC subjects (Fig. 1d). When the ratio between CD56^bright^ and CD56^dim^ NK subsets was calculated, we found that the ratio of CD56^bright^/CD56^dim^ in both LILs and PBMCs was increased significantly in the context of chronic HBV infection, especially in the blood of LC patients (Fig. 1e). These analyses indicated that the frequencies of CD56^bright^ and CD56^dim^ NK subsets were significantly decreased in hepatic and peripheral lymphocytes from HBV patients compared with HC subjects.

We then analysed the expression of NK cell receptors, including natural cytotoxicity receptors (NCR) NKp30, NKp44, NKp46 and NKG2D, and inhibitory receptors NKG2A, CD158a and CD158b; activation markers HLA-DR, CD38 and CD69; and cytotoxic molecules TRAIL, perforin and granzyme A and B, in both hepatic and peripheral total NK cells in the three groups of individuals. Similar to our previous findings^26^,^27^, hepatic and peripheral NK cells displayed elevated expressions of activation receptors (NKp44, NKp46 and NKG2D) and activation markers (TRAIL, HLA-DR, CD38 and CD69), decreased expression of the inhibitory receptors (CD158a and CD158b), and similar levels of expression of perforin and granzyme B in CHB patients compared with HC subjects (Fig. 1f–i). Importantly, the expressions of activation receptors (NKp44, NKp46 and NKG2D), activation markers (TRAIL, HLA-DR and CD69) and cytolytic molecules (perforin and granzyme A) were decreased greatly and the inhibitory receptor CD158b was increased significantly on hepatic NK cells in LC patients compared with CHB patients (Fig. 1f,h). These data indicated that NK cells are highly activated in CHB patients compared with HC subjects; however, when the disease progresses into the LC stage, NK cells were significantly deactivated *in vivo*.

### NK cells from LC patients displayed an impaired anti-fibrotic activity

**Figure 2:**
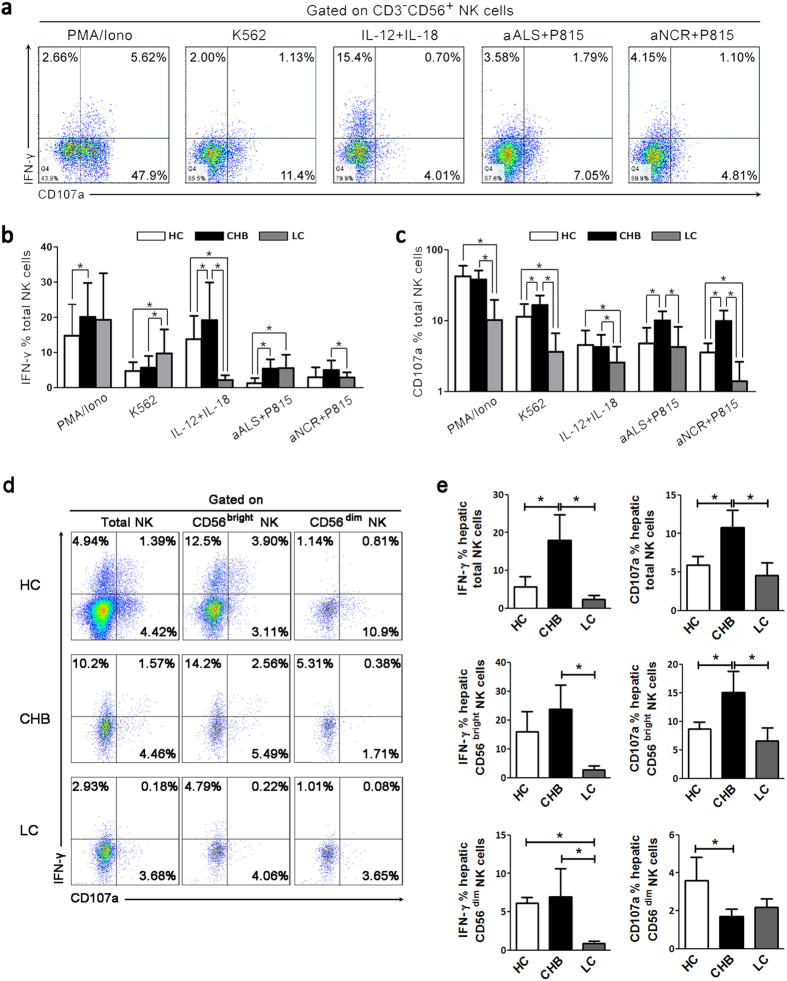
Intrahepatic and peripheral NK cells from LC patients display impaired IFN-γ production and degranulation. (**a**) Representative dot plots showing IFN-γ and CD107a expression on peripheral NK cells among the three groups in response to various stimuli. CD3^−^CD56^+^ NK cells were gated. Values in the quadrants represent the percentages of CD3^−^CD56^+^ NK cells that express IFN-γ and CD107a. (**b,c**) Pooled data showing the percentages of peripheral CD3^−^CD56^+^ NK cells that express IFN-γ (**b**) and CD107a (**c**) from the three groups upon various stimuli (n = 25 for HC; n = 30 for CHB; n = 24 for LC). The data are shown as the means, and the error bars represent the SEM. (**d**) Representative dot plots showing IFN-γ and CD107a expression on intrahepatic total NK cells, CD56^bright^ and CD56^dim^ NK cell subsets among the three groups in response to IL-12 plus IL-18 stimulation. Values in the quadrants represent the percentages of CD3^−^CD56^+^ total and CD56^bright^ and CD56^dim^ NK cell subsets that express IFN-γ and CD107a. (**e**) Pooled data showing the percentages of intrahepatic total NK cells, CD56^bright^ and CD56^dim^ NK cell subsets that express IFN-γ and CD107a from the three groups upon various stimuli (n = 6 for HC; n = 7 for CHB; n = 5 for LC). The data are shown as the means, and the error bars represent the SEM. *P < 0.05, two-tailed, unpaired Student’s t-test.

We next examined whether NK cell deactivation in LC patients influences their anti-fibrosis functions, including cytolysis and IFN-γ production *in vitro*. We first evaluated the ability of peripheral NK cells to produce IFN-γ and CD107a in response to PMA/ionomycin, K562 cells, interleukin (IL)-12 plus IL-18, and redirected cytotoxicity P815 cells bound with a mixture of anti-ALS (anti-CD16) and anti-NCR Abs (anti-NKp30, anti-NKp44 and anti-NKp46) in the three groups (Fig. 2a–c). The analysis indicated that IFN-γ production was increased in CHB and LC patients compared with HC subjects upon these stimulations. Compared with CHB patients, IFN-γ production by NK cells was significantly reduced in LC patients under stimulation by IL-12 plus IL-18 and anti-NCR binding P815 cells (Fig. 2b). CD107a expression by NK cells from CHB patients also increased in response to K562, anti-ALS and anti-NCR binding P815 cells compared with HC subjects; while in the LC patients, CD107a expression by NK cells was markedly decreased compared with that in CHB patients in response to all of these stimulations, even lower than that in HC subjects (Fig. 2c).

Then, we comprehensively dissected the capacity of intrahepatic NK subsets to produce IFN-γ and CD107a in response to IL-12 plus IL-18 using limited liver biopsy from HC donors and CHB and LC patients, respectively. As shown in Fig. 2d, intrahepatic NK cells have also potentials to produce IFN-γ and CD107a under IL-12 plus IL-18 stimulations, especially for CD56^bright^ NK subsets which could produce higher levels of IFN-γ and CD107a than that of CD56^dim^ NK subsets in human livers regardless of disease status. We further found that IFN-γ and CD107a production by intrahepatic total NK cells and CD56^bright^ NK subsets was significantly increased in CHB patients compared with HC subjects upon IL-12/IL-18 stimulations. Compared with CHB patients, IFN-γ and CD107a production by intrahepatic total NK cells and CD56^bright^ NK subsets was significantly reduced in LC patients under IL-12 plus IL-18 stimulation (Fig. 2e). Notably, IFN-γ production by intrahepatic CD56^dim^ NK subsets was significantly reduced in LC patients under IL-12 plus IL-18 stimulation; while CD107a production by CD56^dim^ NK subsets in LC patients is compatible to that of CHB patients (Fig. 2e). These data indicated that both intrahepatic and peripheral NK cell activity was severely impaired when CHB disease progressed into the LC stage.

**Figure 3:**
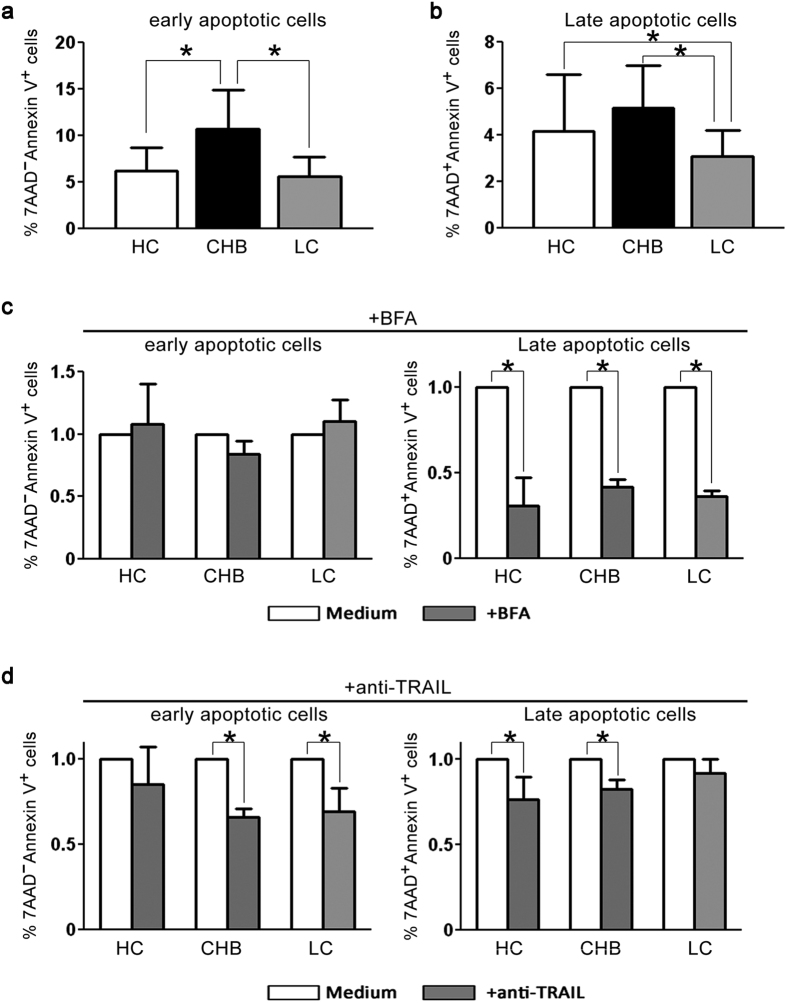
The impaired anti-fibrotic function of NK cells from LC patients against HSCs was dependent on perforin and TRAIL pathway. (**a,b**) Pooled data showing the percentages of apoptotic LX-2 cells (an HSC cell line) by purified NK cells from the three groups at an E: T ratio of 10: 1 (n = 6 for each group of subjects). The percentages of 7-AAD^−^Annexin V^+^ early (**a**) and 7-AAD^+^Annexin V^+^ late apoptotic cells (**b**) are shown. (**c**,**d**) Perforin and TRAIL pathways are involved in the anti-fibrotic activity of NK cells in LC patients. Blockade of perforin using BFA (**c**) and TRAIL (**d**) decreased early and late apoptosis of LX2 cells significantly in CHB and LC patients (n = 6 for each group of subjects). The data are shown as the means, and the error bars represent the SEM. *P < 0.05, two-tailed, paired Student’s t-test.

Finally, we determined the capacity of NK cell killing of LX2 targets (an HSC line). To exclude other cells such as CD8 T cells to kill HSCs, we used isolated NK cells to co-culture with LX2 cells *in vitro* and compared the differences in NK cell killing effects in the three groups of subjects. As illustrated in Fig. 3a,b, purified NK cells from CHB patients could induce more 7-AAD^−^ Annexin V^+^ early apoptotic LX2 cells but comparable 7-AAD^+^Annexin V^+^ late apoptotic LX2 cells compared with those from HC subjects. Importantly, this capacity of NK cells to lyse LX2 targets was significantly lower in LC patients than in CHB patients (Fig. 3a,b). We further investigated which pathway is possibly involved the killing activity of NK cells. As illustrated in Fig. 3c,d, addition of BFA (Brefeldin A, which blocks the perforin-mediated pathway) reduced late apoptosis but not early apoptosis of LX2 cells in these subjects; while blockade of TRAIL using anti-TRAIL antibodies significantly decreased both early and late apoptosis of LX2 cells. Thus, compared with NK cells from CHB patients, NK cells from LC patients displayed a general impairment in the anti-fibrotic activity, reflected in the reduced degranulation, IFN-γ production and killing capacity of LX2 cells, which was partially dependent on the perforin and TRAIL pathways.

### Increased TGF-β impaired the anti-fibrotic activity of NK cells in LC patients

**Figure 4:**
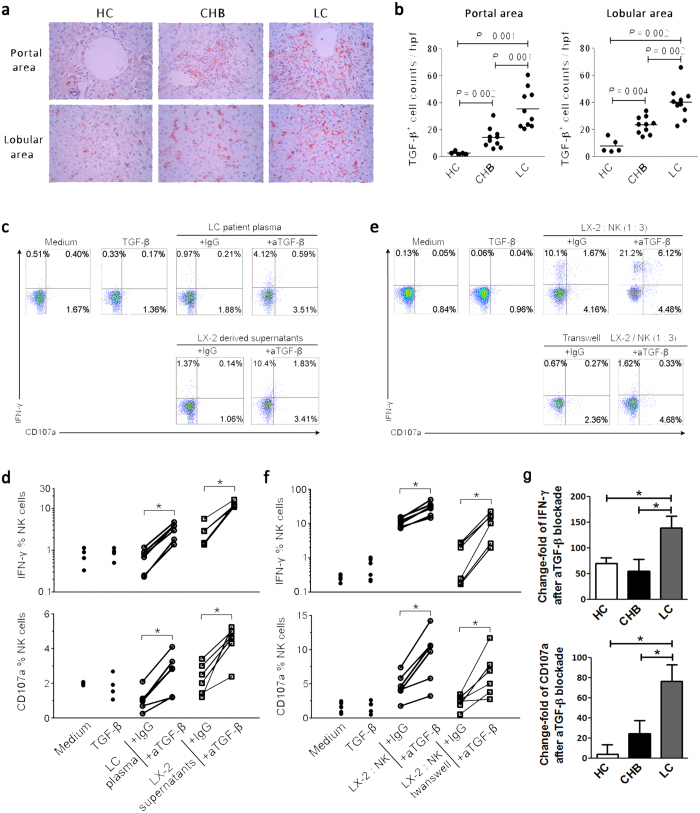
Impaired NK anti-fibrotic functions are dependent on TGF-β in LC patients. (**a**) Representative IHC staining of hepatic TGF-β (red) in the portal and lobular areas from HC, CHB and LC subjects. (**b**) The quantitative analysis of TGF-β^+^ cells in the portal and lobular areas in the livers of the enrolled subjects. Each dot represents one individual. The horizontal lines indicate the mean values. *P*-values shown in the figures are based on two-tailed, unpaired Student’s t-test; hpf, high-power field. (**c,e**) Representative dot plots depicting IFN-γ and CD107a expression on peripheral NK cells from six HC subjects in response to plasma from LC patients or LX2 cell-derived supernatants (**c**), and LX2 cells co-culture or transwell assay (**e**). CD3^−^CD56^+^ NK cells were gated. Values in the quadrants represent the percentages of CD3^−^CD56^+^ NK cells that express IFN-γ and CD107a. (**d,f**) Blockade of TGF-β enhanced the production of CD107a and IFN-γ by NK cells significantly in LC plasma or LX2-derived supernatants (**d**), and LX2 cell culture or with transwell assay (**f**). (**d**,**f**) Each dot represents one individual. *P < 0.05, two-tailed, paired Student’s t-test. (**g**) Pool data indicated that anti-TGF-β treatment has more capacity to restore the production of CD107a and IFN-γ by NK cells from LC patients than that from HC and CHB individuals. Peripheral NK cells from HC (n = 4), CHB (n = 4) and LC (n = 8) individuals were co-cultured with LX-2 cells *in vitro* in the presence of IgG and anti-TGF-β antibodies. The change fold of CD107a or IFN-γ production by NK cells was calculate according to formula: (anti-TGF-β treatment - IgG) / IgG. *P < 0.05, two-tailed, unpaired Student’s t-test.

A previous study indicated that elevated TGF-β could suppress the anti-fibrotic function of NK cells in end-stage liver fibrosis in mouse models^23^. Therefore, we asked whether and how TGF-β inhibits anti-fibrotic activity of NK cells in HBV-infected LC patients. We first examined TGF-β expression in the liver of three groups of subjects using immunohistochemical staining (Fig. 4a). A few, scattered TGF-β^+^ cells were detected in the portal and lobular regions of HC livers, while CHB and LC patients displayed significant numbers of TGF-β^+^ cells in the portal and lobular areas, with a greater number in the LC patients than in the CHB patients (Fig. 4b). We also detected plasma TGF-β levels in three groups of subjects and found that TGF-β concentration in plasma of LC patients was significantly lower than that in HC subjects and CHB patients (Supplemental Fig. 1).

We then investigated whether the elevated levels of hepatic TGF-β drive the impairment of NK cell anti-fibrotic activity. As shown in Fig. 4c, purified NK cells displayed similar levels of production of CD107a and IFN-γ when incubated with medium, TGF-β, plasma from LC patients or LX-2 cells-derived supernatants; however, blockade of TGF-β using anti-TGF-β antibodies significantly enhanced the production of CD107a and IFN-γ by NK cells when incubated with LC plasma and supernatants. Pooled data confirmed this observation (Fig. 4d). By contrast, plasma from LC patients and supernatants from cultured LX2 cells failed to activate NK cells, and blockade of TGF-β could not reduce the CD38 and HLA-DR expression on NK cells *in vitro* (Supplemental Fig. 2). Interesting, when the plasma from HC and CHB subjects was used to co-culture with NK cells, blockade of TGF-β failed to restore the production of CD107a and IFN-γ by NK cells (Supplemental Fig. 3).

It is unclear whether LX2-derived TGF-β could mediate the NK cells’ functional suppression. As shown in Fig. 4e, co-cultured LX-2 cells could stimulate approximately 6% of peripheral NK cells from LC patients to produce CD107a and approximately 12% of NK cells to produce IFN-γ. Separating NK cells and LX2 cells using a trans-well device largely reduced the stimulation of NK cells by LX2 cells. Importantly, we found that the blockade of TGF-β further increased CD107a and IFN-γ production by NK cells from LC patients in the co-culture system by more than 2-fold. This observation was further confirmed by further assays (Fig. 4f). Similarly, co-cultured LX-2 cells could also stimulate peripheral NK cells from both CHB and HC subjects to produce IFN-γ and CD107a (data not shown). Notably, although anti-TGF-β treatment could also increase IFN-γ and CD107a production by NK cells from HC and CHB subjects in the co-culture system, the increased folds were significantly lower than that using NK cells from LC patients (Fig. 4g), suggesting that NK cells from LC patients may have higher sensitivity to TGF-β than those NK cells from CHB and HC subjects. Taken together, these data indicated clearly that although NK cells could kill HSCs in a cell-to-cell contact manner, the increased levels of TGF-β, most likely from activated HSCs, significantly suppressed the anti-fibrotic activity of NK cells from LC patients.

### Intrahepatic NK cells and HSCs interact directly *in vivo* in LC patients

**Figure 5:**
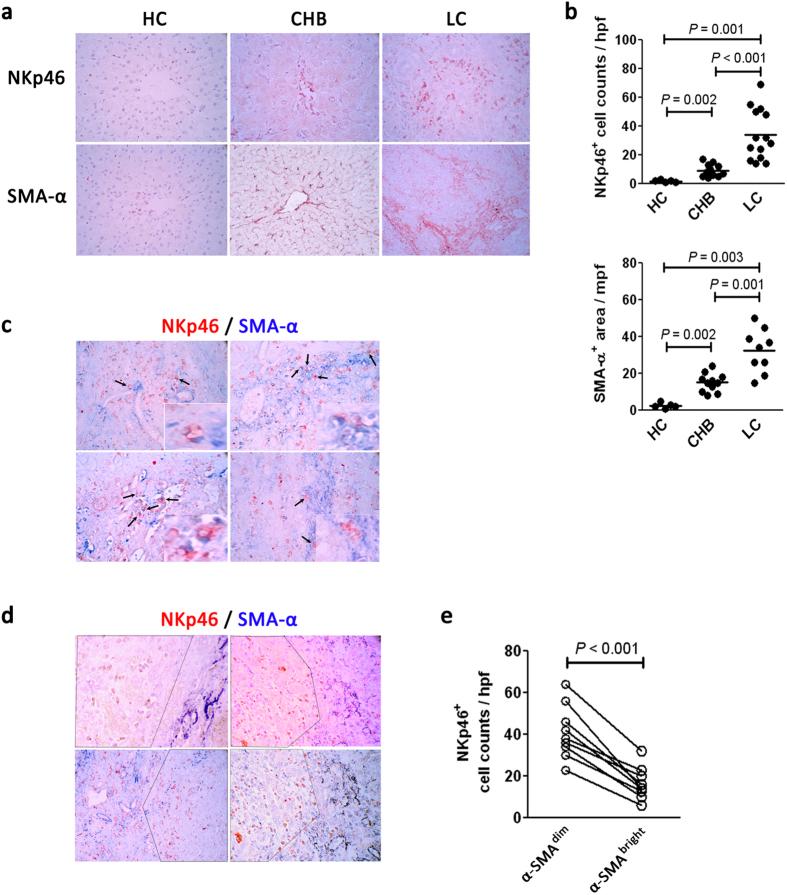
The spatial interaction between hepatic NK cells and HSCs *in vivo* in LC patients. (**a**) Representative IHC staining of NKp46 and α-SMA in the livers of HC, CHB and LC subjects. (**b**) The quantitative analysis of NKp46^+^ cells and SMA-α^+^ cells in the livers of enrolled subjects. Each dot represents one individual. The horizontal lines indicate the mean values. *P*-values shown in the figures are based on two-tailed, unpaired Student’s t-test; hpf, high-power field. (**c**) Representative double staining of NKp46 (red) and α-SMA (blue) indicating the close proximity of NK cells to HSCs in mild fibrotic areas in the livers of four HBV-infected LC patients. The inset figure was magnified to indicate the close interaction of NK cells and HSCs in the livers of LC patients. (**d**) Representative double staining of NKp46 (red) and α-SMA (blue) indicating their separate distribution in severe cirrhotic regions in four HBV-infected advanced LC patients. The dashed line separates the picture into α-SMA^bright^ and α-SMA^dim^ areas. (**e**) The quantitative analysis of NKp46^+^ cells in α-SMA^bright^ and α-SMA^dim^ areas in the livers of LC patients. Each dot represents one individual. *P*-values shown in the figures are based on two-tailed, unpaired Student’s t-test.

The above findings prompted us to examine the spatial distribution of hepatic NK cells and HSCs in livers from HC and HBV-infected patients. Immunohistochemical staining of NKp46 (uniquely expressed by resting and activated by NK cells) and α-SMA^+^ (a marker of activated HSCs) showed that few NKp46^+^ cells and α-SMA^+^ cells were present in the livers of healthy donors. In contrast, NKp46^+^ cells and α-SMA^+^ cells were observed frequently in the livers of HBV-infected subjects, with a greater number in LC patients compared with CHB patients (Fig. 5a,b).

We further examined the co-localization of NKp46 and α-SMA expression in the livers of HBV-infected LC patients. As illustrated in Fig. 5c,d, along the septa of the lobular area where fibrosis is mild, NK cells were frequently located in direct proximity to the α-SMA^+^ cells in LC patients. Alternatively, in some significantly fibrotic regions of LC patients, NKp46^+^ NK cells were found to be distributed separately with α-SMA^+^ HSCs: few NKp46^+^ cells were observed in α-SMA^bright^ areas; by contrast, in α-SMA^dim^ regions, more NKp46^+^ NK cells were detected in the livers of LC patients. The quantitative analysis further confirmed that the NKp46^+^ NK number in the α-SMA^dim^ region was significantly higher than that in the α-SMA^bright^ regions in the livers of LC patients (Fig. 5e). Thus, hepatic NKp46^+^ NK cells accumulated in mild fibrotic regions and directly interacted with α-SMA^+^ HSCs in HBV-infected LC patients. By contrast, in significant fibrotic areas, NKp46^+^ NK cells were more depleted in α-SMA^bright^ region, but enriched in α-SMA^dim^ region, suggesting a possible outcome of the cross-interaction between hepatic NK cells and HSCs in the livers of these patients.

### NK cell emperipolesis is mediated by HSCs in a TGF-β dependent manner

**Figure 6:**
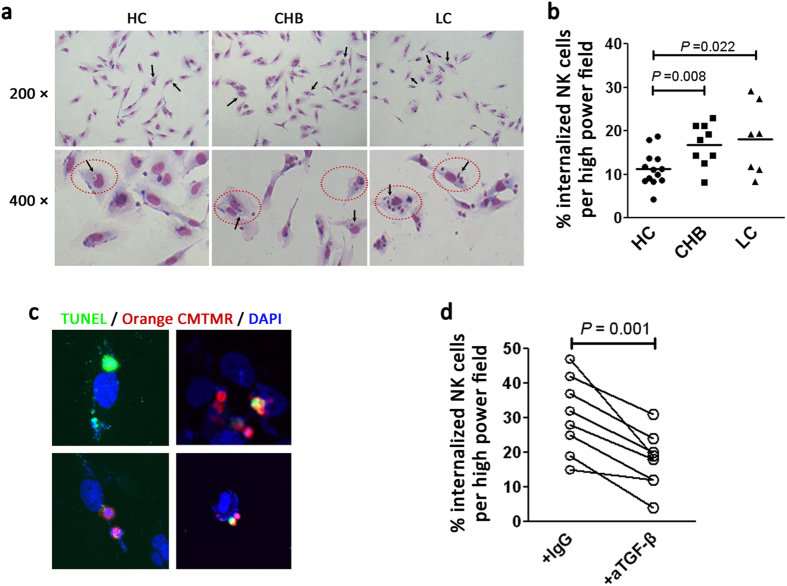
NK cell emperipolesis is mediated by HSCs in a TGF-β dependent manner. (**a**) Representative Giemsa staining indicating the emperipoletic cell-in-cell structures formed between LX-2 cells and purified NK cells from HC, CHB and LC subjects. Internalized NK cells in the cytoplasm of HSCs formed a heterotypic cell-in-cell structure (gated by red dashed ovals), leading to multinucleation of HSCs (black arrow). (**b**) Pooled data showing the ratio of emperipoletic NK cells by HSCs in the three enrolled groups. Each dot represents one individual. The horizontal lines indicate the mean values. *P*-values shown in the figures are based on two-tailed, unpaired Student’s t-test. (**c**) The internalized NK cells by HSCs appear apoptotic under immunofluorescence staining. NK cells from LC patients were pre-stained with CellTracker Orange CMTMR and then co-cultured with LX2 cells, before being subjected to TUNEL staining. CellTracker Orange CMTMR showed the NK/HSC cytoplasm. DAPI stained the nuclei; x630 magnification. (**d**) Pooled data showing the ratio of emperipoletic NK cells by HSCs in the presence of TGF-β or isotype IgG antibody. Each dot represents one individual. *P*-values shown in the figures are based on two-tailed, paired Student’s t-test.

A previous study demonstrated that HCV-derived NK cells contacted cultured human HSCs^12^ directly and were subsequently engulfed by HSCs^28^. In view of this observation, we investigated the outcome of the close contact between hepatic NK cells and HSCs. *In vitro*, we co-cultured purified NK cells and LX-2 cells, and found that NK cells were internalized into the LX-2 cells to form a cell-in-cell structure, which was previously termed as “emperipolesis”^29^. The emperipolesis, based on the entry of NK cells into LX2 cells, was characterized by a halo around the nucleus of NK cells upon Giemsa staining (Fig. 6a).

We then investigated whether NK cells from HC subjects, CHB and LC patients displayed differential levels of emperipolesis in LX-2 cells *in vitro*. Few NK cells from HC subjects could enter LX2 cells; while NK cells from CHB and LC patients displayed a greater potential to be internalized into LX-2 cells (Fig. 6a). Notably, NK cells from LC patients were more likely to form multi-nuclear cell-in-cell structures compared with those from CHB patients and HC subjects; while NK cells from HC subjects and CHB patients only form few mononuclear cell-in-cell structure (gated with red dashed ovals, Fig. 6a). The statistical analysis indicated that the percentages of internalised NK cells were significantly higher in CHB and LC patients compared with HC subjects (Fig. 6b). These data suggested that LX2 cells internalise NK cells from LC patients more easily.

We then investigated the fate of the NK cells internalized by LX2 cells. We pre-labelled NK cells from LC patients using CellTraker Green CMTMR (5-(and-6)-(((4-chloromethyl) benzoyl)amino)tetramethylrhodamine) (mixed isomers) and co-cultured them with LX2 cells. After 6 hours, the cells were collected and stained with terminal deoxynucleotidyl transferase (TdT) dUTP Nick-End Labelling (TUNEL) and 4′,6-diamidino-2-phenylindole (DAPI). Merged images showed that the internalized NK cells exhibited typical apoptotic DNA fragmentation in 4 representative vision fields (TUNEL positive, Fig. 6c; Supplemental Fig. 4). The data suggested rapid apoptosis of NK cells inside LX2 cells by emperipolesis, which may indicated a mechanism underlying failure of hepatic NK cells to anti-fibrosis.

Finally, we determined whether the TGF-β pathway is associated with NK cell emperipolesis by LX2 cells. Anti-TGF-β antibodies or isotype IgG were added into the co-culture medium *in vitro*. Blockade of the TGF-β pathway reduced NK cell emperipolesis within LX2 cells significantly (Fig. 6d). These data supported the notion that HSCs internalize NK cells in a TGF-β-dependent manner.

### The restoration of anti-fibrotic activity of NK cells is associated with liver fibrosis remission in LC patients

**Figure 7:**
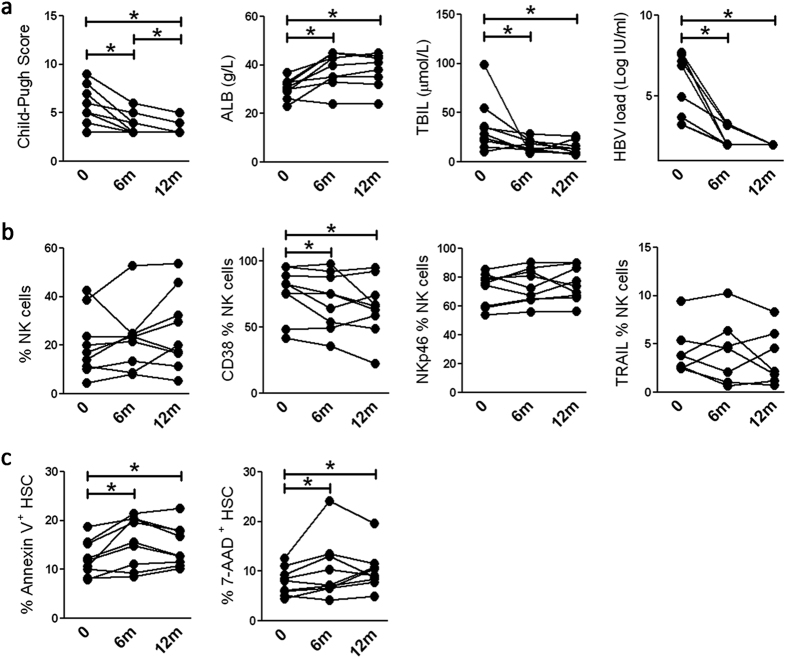
The restoration of anti-fibrotic activity of NK cells is associated with improved liver fibrosis in LC patients. Long-term observation of clinical parameters (**a**), including Child-Pugh scores, TBIL, ALB and HBV load; several key markers of NK cells (**b**), including NK percentages and the expression of CD38, NKp46 and TRAIL on total NK cells; and NK killing capacity against LX-2 cells (**c**), indicated by apoptosis and death of HSCs in LC patients accepting antiviral treatment. Each dot represents one individual. *P < 0.05, two-tailed, paired Student’s t-test.

Finally, we analysed the associations of peripheral NK cells’ anti-fibrotic activities with liver fibrosis restoration in LC patients undergoing clinical treatment. As shown in Fig. 7a, LC patients accepting clinical antiviral treatment displayed a good outcome, indicated by significantly decreased Child-Pugh scores and total bilirubin (TBIL) concentration, increased serum albumin (ALB) levels and undetectable HBV load. Meanwhile, NK frequency, NKp46 and TRAIL expression by total NK cells showed a decreasing trend with the continuing remission of liver cirrhosis. The expression of the activation marker CD38 on NK cells was significantly decreased by long-term antiviral treatment (Fig. 7b). The cytolytic potential of NK cells to kill LX-2 cells was also enhanced by clinical treatment, especially at 6 month since the onset of treatment (Fig. 7c). These data indicated that restoration of the anti-fibrotic activities of NK cells is associated with remission of liver fibrosis in LC patients.

## Discussion

**Figure 8:**
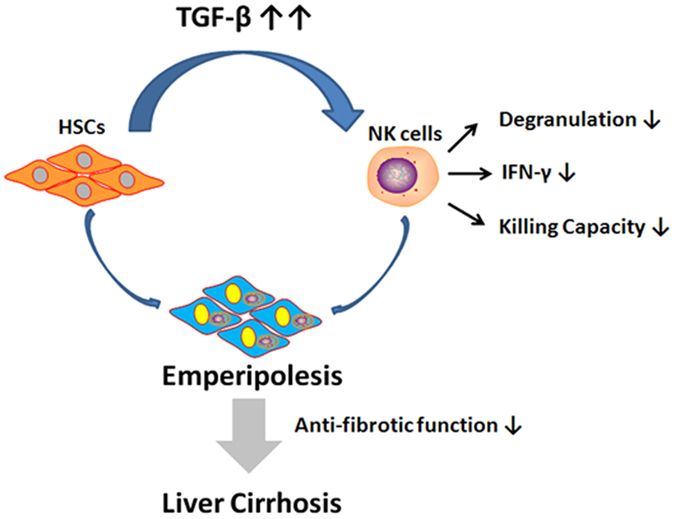
A model depicting the impact of HSCs on NK cell anti-fibrotic functions in LC patients with HBV infection. In advanced liver fibrosis, activated HSCs generally produce a large amount of TGF-β that might suppress NK cell anti-fibrotic functions, such as degranulation, IFN-γ production and killing activity against HSCs. The HSCs might then internalize these functionally impaired NK cells in an emperipolesis-dependent manner, thus promoting the progression of liver fibrosis.

NK cells displayed anti-fibrotic activity by killing activated HSCs and producing IFN-γ in mouse models^11^,^12^,^14^,^15^,^16^,^17^,^18^,^23^,^30^,^31^. However, the immune status of intrahepatic NK cells and the potential regulatory role of HSCs on them especially in LC patients with chronic HBV infection, remain unclear. The present study demonstrated that hepatic NK cells showed a numerical decrease, phenotypic deactivation, impaired anti-fibrotic activity and close spatial interaction with HSCs in HBV-infected LC patients compared with CHB patients and HC subjects. We also found that activated HSCs in LC patients induced apoptosis of hepatic NK cell in a cell emperipolesis manner that is partially dependent on TGF-β (Fig. 8). These findings addressed the potential impact of activated HSCs on NK cell anti-fibrosis function in LC patients with chronic HBV infection.

Deactivation is a prominent characteristic of hepatic NK cells in LC patients compared with CHB patients. The present study provided several lines of evidence to support this notion. First, activation receptor (NKp30-, NKp44-, NKp46- and NKG2D)-expressing NK cells were reduced, whereas inhibitory receptor (CD158a/b)-expressing NK cells were increased in the livers of HBV-LC patients compared with CHB subjects. Second, the expression levels of activation markers HLA-DR and CD69 on hepatic NK cells from HBV-infected LC patients were significantly lower than those from CHB patients. Third, the expressions of several functional molecules, such as TRAIL, perforin and granzymes, were also decreased in hepatic NK cells from HBV-infected LC patients compared with CHB patients. Fourth, the deactivation of NK cells in HBV-infected LC patients was also mirrored by their functional decrease in CD107a degranulation and IFN-γ production, as well as cytolytic activities against HSCs in response to various stimulators *in vitro*. Particularly, hepatic CD56^bright^ NK cells from LC patients, a population of NK subsets representing nearly 50% of NK cells in the human liver, displayed more severe functional impairment than that from CHB patients. Finally, with the clinical improvement of HBV-infected LC patients, the activation status of NK cells was restored and the impaired cytolytic activity of NK cells was recovered. These data clearly demonstrated that in advanced LC patients, NK cells are characterized by deactivation and the loss of anti-fibrosis function compared with those from CHB patients, who generally displayed mild liver fibrosis. Combined with previous reports in mice^23^,^25^, the impaired anti-fibrotic functions of NK cells have been shown to be associated with accelerated progression of liver fibrosis.

It remains unclear which mechanisms mediate the loss of NK cell anti-fibrotic activity in advanced LC patients. We provided one explanation: the increased TGF-β levels in the livers of LC patients might inhibit NK cell cytolytic activity and IFN-γ production, since blockade of TGF-β significantly enhanced NK functions *in vitro*. Indeed, in a mouse model, intermediately activated HSCs could produce more TGF-β and inhibit IFN-γ production of NK cells compared with early-activated HSCs, and blocking TGF-β also restored NK cell killing and IFN-γ production^23^. These data suggested that fully activated α-SMA^+^ HSCs produce a large amount of TGF-β that subsequently deactivates NK cells and inhibits their anti-fibrotic effect, particularly in advanced LC patients. Although TGF-β is involved actively in the inhibition of NK cells prevention of liver fibrosis, two questions remain. First, in addition to HSCs, kupffer cells also play an important role in producing TGF-β during liver fibrogenesis^32^,^33^. IL-17^34^,^35^,^36^,^37^ and IL-22^38^,^39^ could also exert pro-fibrosis effects in chronic liver injury through recruiting lymphoid cells to infiltrate into the livers. Second, NK cells from LC patients may have higher sensitivity to TGF-β than those NK cells from CHB and HC subjects because anti-TGF-β treatment had more potentials to restore NK cell function in LC patients than that of HC and CHB individuals. Therefore, future studies are required to determine which mechanisms negatively regulate NK cell functions through TGF-β or induce high sensitivity of hepatic NK cells to TGF-β in various stages of liver fibrosis.

We also provided evidence of how the activated HSCs-derived TGF-β inhibits the anti-fibrotic function of NK cells in LC patients. HSCs-derived TGF-β might suppress IFN-γ and degranulation of NK cells, both of which have been demonstrated to be important anti-fibrotic factors mediated by NK cells *in vivo* and *in vitro*^11^,^12^,^17^,^22^,^23^. The present study indicated that the cytolytic activity of NK cells against HSCs mainly depends on perforin and TRAIL, because blockade of the perforin and TRAIL pathways partially reduced the apoptosis of LX2 cells induced by NK cells. In addition perforin and TRAIL, other pathways, including the RAE-1/NKG2D^11^,^18^, NKp46^14^,^20^ and NKp30 pathways^21^ or the inhibitory NK receptor^12^,^15^, are also involved in the killing of activated HSCs by NK cells. Indeed, elevated TGF-β might suppress the anti-fibrotic function of NK cells by down-regulating NKG2D and 2B4 surface expression^23^,^25^,^40^. A future study should address which pathways are involved in the TGF-β-mediated suppression of NK cell anti-fibrotic function in advanced LC.

Importantly, the present study demonstrated that the activated HSCs have the potential to internalize functionally suppressed NK cells, further leading to their apoptosis. NK cells were observed in close proximity to HSCs in mild fibrotic areas, but were distributed separately in severe cirrhotic regions in advanced LC patients. This anatomically differential distribution indicated a complex process and various outcomes of the interaction between hepatic NK cells and activated HSCs within various fibrosis areas, and suggested two possible pathological outcomes: NK cells kill the activated HSCs to prevent liver fibrosis progression or activated HSCs impair NK cell anti-fibrotic function, leading to NK cell death. Our data found that NK cells from LC patients were internalized into activated HSCs *in vitro* and blockade of TGF-β significantly reduced this phenomenon. This internalisation was first named as “emperipolesis” in 1956 by Humble *et al*.^29^, and was characterized by the heterogeneous cell-in-cell structure indicated by a halo around the nucleus of lymphocytes. Emperipolesis has been observed in autoimmune hepatitis (AIH), CHB, drug-induced liver injury (DILI) and hepatitis C^41^,^42^,^43^,^44^ and was included as a characteristic feature of the histopathology of AIH^45^,^46^. In tumours, emperipolesis of immune cells has also been reported in non-neoplastic and neoplastic host cells *in vivo* and *in vitro*^47^, especially for NK cells, which enter into tumour cells and induce self-destruction through activating caspase-3 and DNA fragmentation in an erzin-dependent manner^48^. The consequences of emperipolesis are also debatable. Some studies suggested that lymphocytes might use emperipolesis to kill target cells. Alternatively, target cells may destroy invading lymphocytes as a mechanism to escape immune surveillance^49^. Our study indicated the NK cells are TUNEL^+^ within LX2 cells *in vitro*, which suggested that “suicidal emperipolesis” might be a unique mechanism of NK cell deletion, a process critical for the maintenance of liver fibrosis. Interestingly, a previous study suggested that activated HSCs could phagocytose non-apoptotic lymphocytes, subsequently inducing their inactivation via apoptosis and death inside the HSCs, which was associated with liver fibrosis^28^. Future study requires a thorough investigation of the mechanisms leading to the emperipolesis and phagocytosis of hepatic NK cells by activated HSCs during the process of liver fibrosis and the associated clinical significance.

Taken together, these findings suggested that HSCs have a great impact on hepatic NK cell anti-fibrotic functions via TGF-β, which suppresses NK cells’ killing capacity and IFN-γ production, and induces NK apoptosis through cell emperipolesis in HBV-infected LC patients. Future careful *in vivo* analysis is needed to address several important questions, such as the spatiotemporal details of the NK-HSC interaction, the clinical significance of NK cell emperipolesis by HSCs or other host cells, and the functional roles of the different subpopulations of liver NK cells in the process of liver fibrosis^24^.

## Methods

### Study subjects

Thirty-six CHB patients and 43 HBV-associated LC patients were recruited for this study. All patients were diagnosed according to our previously described criteria^26^,^50^ and were not taking antiviral therapy or immunosuppressive drugs within six months before sampling. Thirty-eight age- and sex-matched healthy individuals were enrolled as health controls. Individuals with concurrent HCV, hepatitis G virus or human immunodeficiency virus infections, autoimmune liver diseases or alcoholic liver disease, were excluded. The study protocol was approved by the ethics committee of Beijing 302 Hospital, and written informed consent was obtained from each subject. Experiments were carried out in accordance with approved guidelines of Beijing 302 Hospital. The basic characteristics of the enrolled subjects are listed in Supplemental Table 1.

PBMCs were isolated from all enrolled subjects. Nine LC patients accepting clinical treatment such as antiviral treatment were followed-up for more than 12 months. Liver biopsies were collected from 29 CHB patients and 18 LC patients, and 13 healthy liver tissue samples were obtained from the healthy donors whose livers were used for liver transplantation. Hepatic necro-inflammation and fibrosis were graded using the Scheuer scoring system. Except for pathological evaluation, liver biopsy specimens were homogenized for the isolation of LILs, and embedded in Tissue Tek for *in situ* immunohistochemical staining.

### Fluorescence activated cell screening (FACS) analysis

All antibodies were purchased from BD Biosciences (San Jose, CA, USA) with the exception of phycoerythrin (PE)-conjugated anti-CD158a, anti-CD158b, anti-NKG2A, anti-NKG2D, anti-NKp30, anti-NKp44, anti-NKp46 and anti-TRAIL antibodies, which were purchased from R&D Systems (Minneapolis, MN, USA). NK subset frequencies and NK receptor expressions were analysed according to previously described protocols^26^,^50^. To detect NK cell activation, PBMCs were incubated with PE-conjugated anti-CD3, APC-conjugated anti-CD56 and PerCP-conjugated anti-HLA-DR, or FITC-conjugated anti-CD69 and anti-CD38. For intracellular staining, cells were permeabilised and stained with the corresponding intracellular antibodies, including those recognising perforin, granzyme A and B. Cells were then analysed using FACSCaliber or FACSCanto II and Flowjo software (TreeStar, Ashand, OR, USA).

### Degranulation of NK cells and IFN-γ detection

Freshly isolated PBMCs (5 × 10^5^) were stimulated directly stimulated with PMA (50 ng/ml) and ionomycin (1 μg/ml), or K562 cells at the effector target ratio of 10:1, or IL-12 (50 ng/ml) in combination with IL-18 (50 ng/ml; Biovision, PA, CA), or were cultured with P815 target cells (at the ratio of 1:1) in the presence of an anti-ALS antibody (0.5 ng/ml, an anti-CD16 FcγR III IgM antibody (Immunological Sciences), or mAbs specific for NKp30, NKp44 and NKp46 in combination (1 ng/ml, respectively; Biolegend). Due to limited liver biopsy, freshly isolated LILs were only stimulated with IL-12 (50 ng/ml) in combination with IL-18 (50 ng/ml). Unstimulated PBMCs served as negative controls. Anti-CD107a and monensin were added directly into the medium. After 6 h of incubation, the cells were collected and stained with surface antibodies and stained intracellularly with anti-IFN-γ.

### NK cell purification and cytolytic killing assay

NK cells were purified using micro-beads according to the manufacturer’s instructions, and subsequently incubated with the HSC cell line (LX2 cells) at the ratio of 10:1 for 6 h in the presence of BFA or anti-TRAIL antibodies. The target cells alone were used as controls. The cells were then stained with 7-aminoactinomycin D (7-AAD, 1 μg/ml) and Annexin V to identify the early and late apoptotic cells, respectively.

### Cell stimulation and transwell assay

LX-2 cells were cultured in medium containing 10% fetal bovine serum (FBS) for 3 days. The supernatants were then collected for subsequent experiments. The freshly isolated PBMCs (2 × 10^6^ cells/ml) were cultured in medium alone or with TGF-β (10 ng/ml) (Pepertech, NJ, USA) or plasma from HC subjects, CHB and LC patients or LX-2-derived supernatants in the presence of anti-TGF-β or IgG isotype control antibodies for 48 h. Alternatively, the PBMCs from HC, CHB and LC individuals were either directly co-cultured with HSCs or separately incubated with HSCs in a trans-well assay, in the presence of anti-TGF-β or IgG isotype control antibodies for 48 h; 6 h before the end of the experiment monensin and CD107a were added into the medium. The lymphocytes were collected and stained with antibodies against surface markers and further subjected to 6 h of degranulation and intracellular IFN-γ detection, as described above. LX-2 cells were collected and stained with 7-AAD and Annexin V to identify the early and late apoptotic cells, respectively.

### Cell-in-cell assay

NK cells were purified from HC, CHB and LC patients using micro-beads (Miltenyi Biotech, Bergisch-Gladbach, Germany), and subsequently incubated with the LX-2 cells at a ratio of 4:1 for 4 h in the presence of anti-TGF-β antibodies or IgG isotype control antibodies. The target cells alone were used as controls. The cells that had undergone emperipolesis were then identified under the microscope and counted. Alternatively, purified NK cells from LC patients were pre-stained CellTracker Orange CMTMR and then co-cultured with LX2 cells for 4 h. The cells were then subjected to TUNEL staining and the nuclei were counterstained with DAPI.

### Immunohistochemical staining

Optimal cutting temperature (OCT) compound embedded liver biopsy cryosections and paraffin-embedded sections (both 5 μm) were incubated with anti-NKp46 (Clone 195314, R&D Systems), anti-SMA-α (Rabbit polyclonal, Abcam) or TGF-β (Rabbit polyclonal, Abcam), respectively, according to our previously described protocols^26^,^38^. Double staining was performed using mouse anti-NKp46 and anti-SMA-α to evaluate the co-location of hepatic NK cells and HSCs. High-powered fields (hpf, 400×) were used to count positive cells. Positive cells were counted in three different fields by two independent observers.

### Statistical analysis

All data were analysed using SPSS 13.0 for Windows software (SPSS Inc., Chicago, IL, USA). Comparisons between various individuals were performed using the unpaired Student’s t-test; while comparisons between the same individual were performed using the Wilcoxon matched pairs *T* test. Correlations between variables were evaluated using the Spearman rank correlation test. For all tests, two-sided *P *< 0.05 was considered significant.

## Additional Information

**How to cite this article**: Shi, J. *et al*. Activated hepatic stellate cells impair NK cell anti-fibrosis capacity through a TGF-β-dependent emperipolesis in HBV cirrhotic patients. *Sci. Rep.* **7**, 44544; doi: 10.1038/srep44544 (2017).

**Publisher's note:** Springer Nature remains neutral with regard to jurisdictional claims in published maps and institutional affiliations.

## Supplementary Material

Suplemental Information
