# Supplementary material for: Activated hepatic stellate cells impair NK cell anti-fibrosis capacity through a TGF-β-dependent emperipolesis in HBV cirrhotic patients
Source: Sci Rep. 2017 Mar 14;7:44544. doi: 10.1038/srep44544 (PMC5349579; doi:10.1038/srep44544)
Supplement: Suplemental Information [file srep44544-s1.pdf]

Activated hepatic stellate cells impair NK cell anti-fibrosis capacity through a TGF-β-dependent emperipolesis in HBV cirrhotic patients

Jijing Shi, Juanjuan Zhao, Xin Zhang, Yongqian Cheng, Jinhua Hu, Yuanyuan Li, Xin Zhao, Qinghua Shang, Yanling Sun, Bo Tu, Lei Shi, Bin Gao, Fu-Sheng Wang, Zheng Zhang

Supplemental information

Supplemental Table 1. Clinical characteristics of enrolled subjects

**Category**	**healthy control (HC)**	**chronic hepatitis B (CHB)**	**liver cirrhosis (LC)**
Cases	38	36	43
Age (years)	29 (21–48)	31 (19–52)	48 (25–68)
Gender (M/F)	28/10	28/8	37/16
Alanine aminotransferase (IU/L)	ND	72 (6–627)	35 (8–218)
Aspartate Aminotransferase (IU/L)	ND	43 (14–481)	47 (15–278)
Serum albumin (g/L)	ND	39 (32–50)	33 (23–44)
Total bilirubin (μmol/L)	ND	14.4 (0.82–414)	26.7 (7.1–181)
Direct bilirubin (μmol/L)	ND	5.4 (1.6–236)	12.9 (4.0–301)
Prothrombin activity (%)	ND	92 (68–190)	66.4 (22–101)
HBeAg positive/negative	ND	18/18	30/23
Serum HBV levels (IU/ml)	ND	3.5×10^7 ($100–4.6 \times 10^9$)	2.8×10^5 ($100–1.1 \times 10^8$)

Data are shown as median and range. ND, no data.

Supplemental Figure Legends

Supplemental Figure 1. Plasma TGF-β levels in HC subjects and CHB and LC patients. Each dot represents one individual. The horizontal lines indicate the mean values. *P*-values shown in the figures are based on two-tailed, unpaired Student's t-test.

Supplemental Figure 2. TGF-β does not influence the activation of NK cells. Representative dot plots depict CD38 and HLA-DR expression on peripheral NK cells from six HC subjects under various culture conditions. $CD3^{-}CD56^{+}$ NK cells were gated. Values in the quadrants represent the percentages of $CD3^{-}CD56^{+}$ NK cells that express CD38 and HLA-DR.

Supplemental Figure 3. Blockade of TGF-β failed to enhance the production of CD107a and IFN-γ by NK cells co-cultured with plasma from HC and CHB patients. Each dot represents one individual.

Supplemental Figure 4. Immunofluorescence staining indicated that NK cells were internalized by HSCs and displayed an apoptotic status in HSCs. NK cells from LC patients (n = 4) were pre-stained with CellTracker Orange CMTMR and then co-cultured with LX2 cells, before being subjected to TUNEL staining. CellTracker Orange CMTMR showed the NK/HSC cytoplasm. DAPI stained the cell nuclei; × 630 magnification.

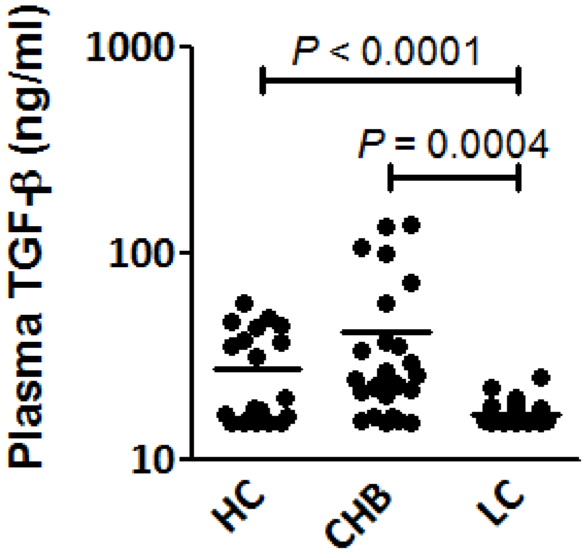

Supplemental Figure-1 (Zhang)

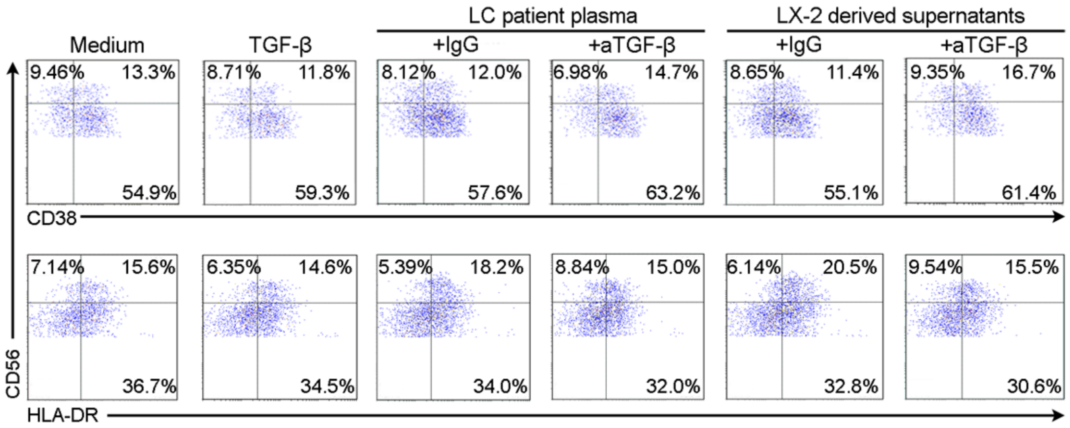

Supplemental Figure-2 (Zhang)

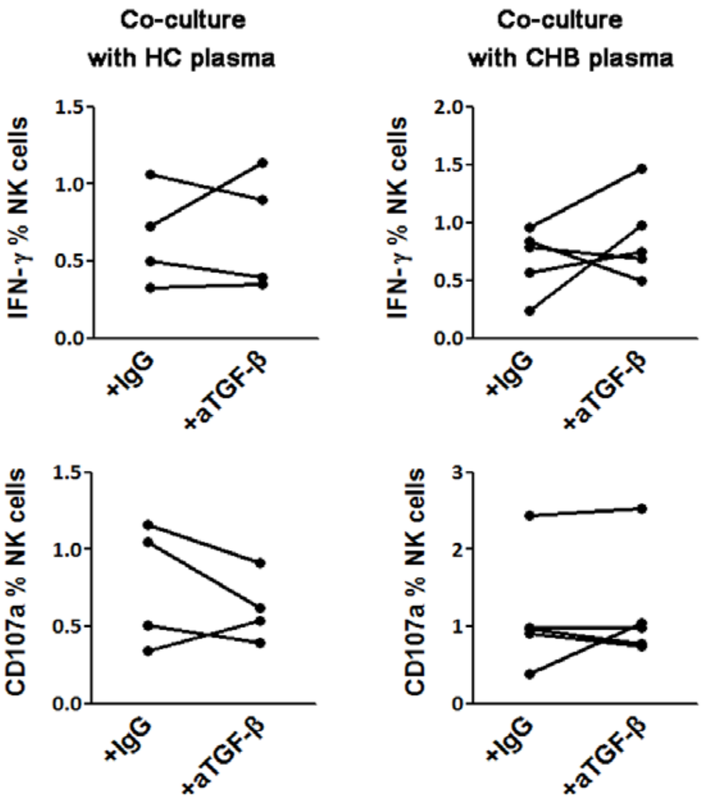

Supplemental Figure-3 (Zhang)

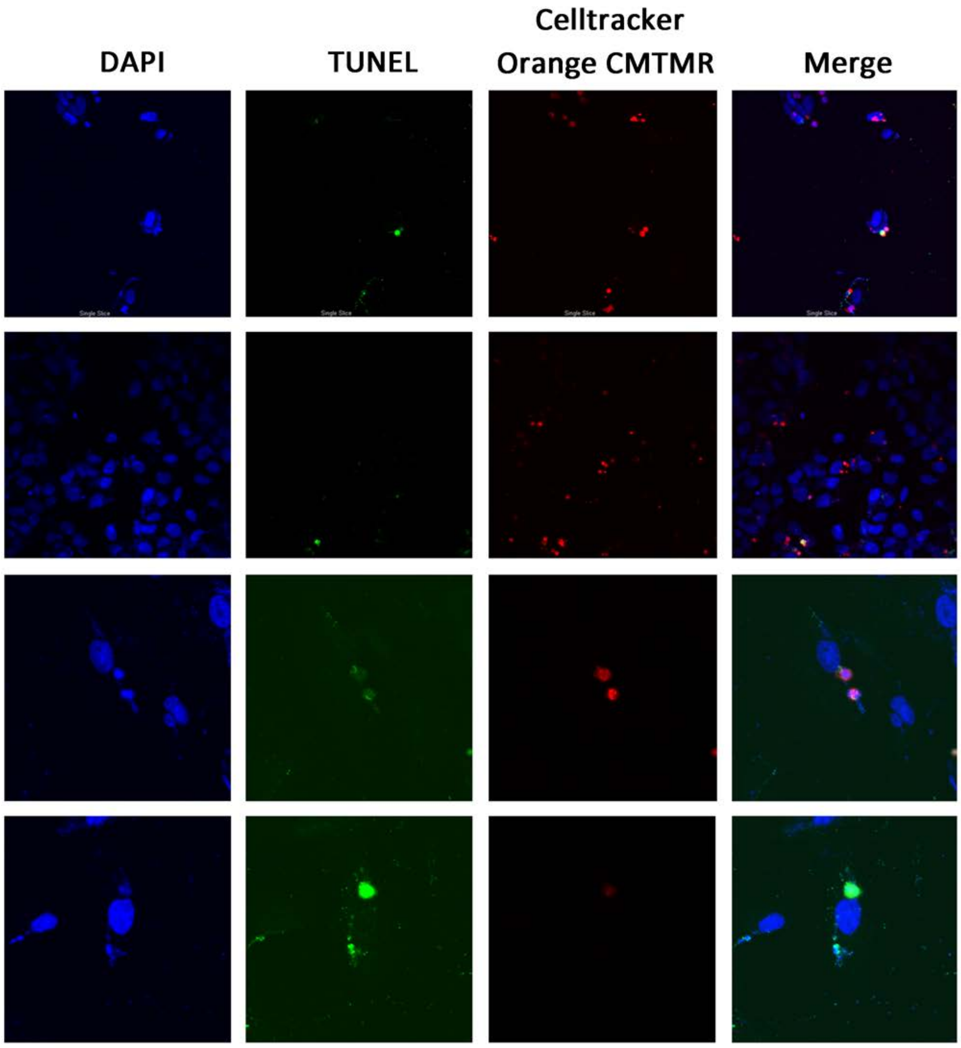

Supplemental Figure-4 (Zhang)
